# Salt Intake Estimation from Urine Samples in South Asian Population: Scoping Review

**DOI:** 10.3390/nu15204358

**Published:** 2023-10-13

**Authors:** Ummay Afroza, Ahmad Khairul Abrar, Abira Nowar, Jubaida Akhtar, Mohammad Abdullah Al Mamun, Sheikh Mohammad Mahbubus Sobhan, Laura Cobb, Nicole Ide, Sohel Reza Choudhury

**Affiliations:** 1 Department of Epidemiology and Research, National Heart Foundation Hospital and Research Institute, Dhaka 1216, Bangladesh; abrar.bd@gmail.com (A.K.A.); abiranowar0695@gmail.com (A.N.); jubaidasa@yahoo.com (J.A.); abdullah.mmcm26@gmail.com (M.A.A.M.); smms_lincoln@yahoo.com (S.M.M.S.); sohel_r_choudhury@hotmail.com (S.R.C.); 2 Resolve To Save Lives (RTSL), New York, NY 10004, USA; lcobb@resolvetosavelives.org (L.C.); nide@resolvetosavelives.org (N.I.)

**Keywords:** salt, sodium, urinary sodium, spot urine, South Asia

## Abstract

The World Health Organization recommended reducing one’s salt intake below 5 g/day to prevent disability and death from cardiovascular and other chronic diseases. This review aimed to identify salt estimation at the population level in South Asian countries, namely Afghanistan, Bangladesh, Bhutan, India, Nepal, Pakistan, and Sri Lanka. We searched electronic databases and government websites for the literature and reports published between January 2011 and October 2021 and also consulted key informants for unpublished reports. We included studies that assessed salt intake from urinary sodium excretion, either spot urine or a 24 h urine sample, on a minimum of 100 samples in South Asian countries. We included 12 studies meeting the criteria after screening 2043 studies, out of which five followed nationally representative methods. This review revealed that salt intake in South Asian countries ranges from 6.7–13.3 g/day. The reported lowest level of salt intake was in Bangladesh and India, and the highest one was in Nepal. The estimated salt intake reported in the nationally representative studies were ranging from 8 g/day (in India) to 12.1 g/day (in Afghanistan). Salt consumption in men (8.9–12.5 g/day) was reported higher than in women (7.1–12.5 g/day). Despite the global target of population salt intake reduction, people in South Asian countries consume a much higher amount of salt than the WHO-recommended level.

## 1. Introduction

Premature death resulting from non-communicable diseases (NCDs) is increasing over time, with NCDs accounting for about 71% (41 million) of deaths worldwide [1]. Cardiovascular diseases (CVDs) is the major cause of death globally, accounting for 30% of all global fatalities and 44% of all NCD-related deaths [2]. Based on the Global Burden of Disease (GBD) 2019 estimate, the total CVD prevalence was 523 million and there were 18.6 million CVD deaths [3]. CVDs are regarded as the major consequence of dietary risks. Annually, almost 8 million deaths and 188 million disability-adjusted life years (DALYs) are attributed to dietary risks [3]. Excess sodium consumption is one of the major dietary risk factors. Due to excess dietary sodium consumption, there were over 1.89 million deaths and over 44.87 million DALYs [4]. High sodium consumption is related to hypertension development which is attributed to two major CVDs, stroke and coronary heart disease. Moreover, high sodium consumption is also found to be associated with obesity, osteoporosis, renal disease, and gastric cancer [5,6]. In addition to health problems, there is also a significant social, developmental, and financial loss to families affected by hypertension from caregiving and premature mortality [7]. The growing burden of the consequences of salt consumption must be reduced through lifestyle changes and strengthening the country-specific food policy environment.

The World Health Organization (WHO) has recognized salt reduction as a “best buy”, or one of the most cost-effective and feasible interventions to reduce the burden of NCDs [8]. WHO member states have committed to lowering the mean population salt intake by 30% by the year 2025 [9]. The global sodium intake estimated in 2019 was 4.3 g/day (10.8 g/day salt) and the second highest was reported from the Southeast Asian region, which was 3.9 g/day (9.8 g/day salt) [9]. Processed foods and meals made outside the house account for over 75% of salt in many high-income nations’ diets [10]. In contrast, discretionary salt use is the major source of dietary sodium in many low-and middle-income nations. As a result of the rapid urbanization and expansion of food industries, South Asian countries have undergone an epidemiological transition, moving from their traditional diets to diets high in processed foods [11]. The GBD study of 2017 showed that the average salt intake of the South Asian population is around 10.16 g/day, which is more than double the WHO-recommended daily salt intake level [2]. Estimating dietary salt intake at the population level is challenging and resource intensive, particularly in collecting 24 h urinary sodium excretions, which is considered the Standard method for measuring the salt consumption level, but many countries lack the resources to conduct this method. Another method, the spot urinary estimation of sodium, is less accurate, but there is evidence that spot urine samples can be used to provide snapshot estimates of the mean population salt intake at a specific point in time. Thus, in order to provide a general snapshot of the regional situation, it is useful to include studies that used spot urine collections [12,13]. Other methods, such as applying the Kawasaki, Tanaka, and INTERSALT formulas to estimate the 24 h urine or monitor changes in the population salt intake, are not suitable, as they systematically underestimate higher salt intakes and overestimate lower salt intakes [12].

In order to effectively monitor the policies and strategies undertaken, countries need a reliable estimate of the population’s mean salt intake level. Various methods have been used by countries to estimate the population’s salt intake, ranging from dietary recall questionnaires to the gold standard estimation by multiple 24 h urinary sodium measurements. Comparative documentation on the reliable estimate of the population-based salt intake of different countries will enhance the expansion of salt reduction initiatives. There is limited comparative evidence on the current salt consumption situation of South Asian countries. A recent review conducted by Ghimire et al. identified all the studies reporting the salt intake by South Asian countries, including studies on specific sub-populations (i.e., hospital-admitted patients) as well as studies that estimated salt intake by any method, which found a range of salt intake from 4.4 (4.1–4.7) g/day to 17.0 (13.8–20.2) g/day [14]. However, given the wide inclusion criteria that likely captured non-nationally representative populations and some studies using unreliable estimating methods, there remains some uncertainty as to the range of reliable estimates for population-level salt consumption across the region. This review is aimed to build on the Ghimire et al. study to compile the current reliable population-based estimates of salt intake in South Asian countries. This document could be used in awareness-raising and salt reduction advocacies providing evidence on the high salt intake by these regional countries.

## 2. Methods

In this study, we adopted the common framework of a scoping review—(1) Identifying the research question; (2) Identifying relevant studies; (3) Selection of studies; (4) Data charting; (5) Collating, summarizing, and reporting the results; and (6) Consultation with other reviewers (optional and was not adopted in this research).

### 2.1. Stage I: Identifying the Research Question

For this review, the research question was: What is the current state of salt consumption level in South Asia identified by the original research?

### 2.2. Stage II: Identifying Relevant Studies

A thorough search of the literature for relevant studies was done using the appropriate keyword sets. We searched for both academic articles and the grey literature (Table 1 for list of databases), including reference lists. Every available keyword was used (Table 2 for list of keywords) using boolean operators: OR” and “AND” accordingly.

### 2.3. Stage III: Study Selection

**Figure 1:**
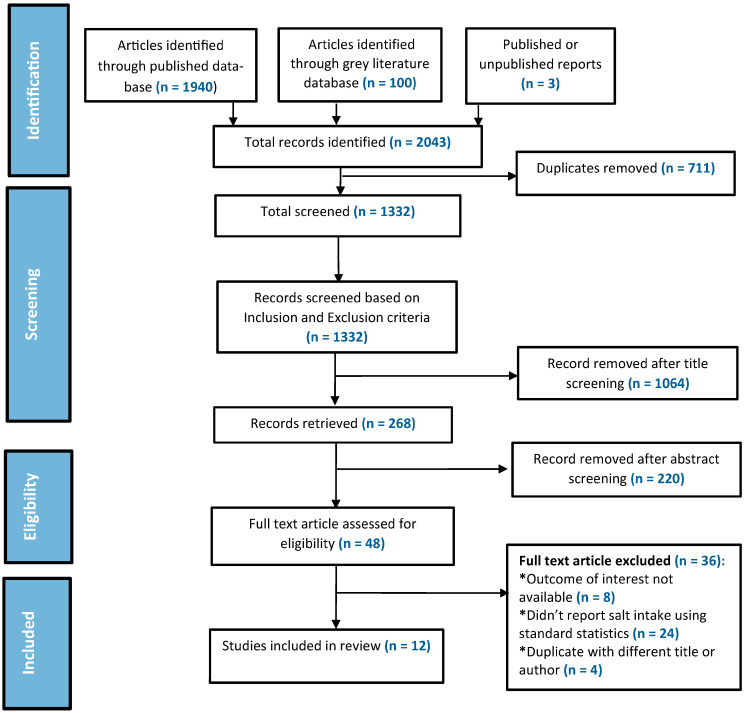
PRISMA-scoping review flow chart for study selection process.

Extensive searches of multiple databases generally reveal many repetitive or irrelevant studies requiring a disposal mechanism. Thus, we removed any duplicate entries. Additionally, we developed relevant inclusion and exclusion criteria for our research questions (Table 3). We defined South Asia as SAARC nations (Afghanistan, Bangladesh, India, Nepal, Pakistan, Sri Lanka, Bhutan, and Maldives) and only included studies from these countries. We included studies where the population’s salt intake was estimated using urinary samples, either spot or 24 h, or both. We excluded studies that did not report the salt intake by standard statistics or only reported percentages. Additionally, we excluded studies conducted only on special populations (e.g., hospital-admitted). We only selected studies that were published in English. Our study selection procedure consisted of two phases. First, we reviewed the article titles and abstracts to ensure they fulfilled our inclusion criteria. Second, we obtained full texts of the articles from the first phase that we had decided to keep, reviewed them, and then removed any that were not relevant to the research question. Figure 1 shows the study selection process as a flowchart.

### 2.4. Stage IV: Data Charting

The following data were gathered from the selected articles: author(s), year of publication, type of study, study population, method of estimating salt, and amount of salt intake mentioned. These data were graphed in Microsoft Excel. The search strategy is presented below through the PRISMA diagram (Figure 1).

### 2.5. Stage V: Reporting Results

Scoping studies do not seek to assess the quality of the literature; rather, they seek to create a description of what research exists or to design a thematic structure to provide a comprehensive picture of research in a certain topic area. We attempted to order the included articles according to the study methods.

### 2.6. Units

In this review, we expressed the amount of salt in g/day. Urinary sodium excretion is often expressed as milliequivalent or millimolar sodium. In many studies reviewed here, authors converted urinary sodium to dietary salt using relevant formulas. In some of the articles included in this review, dietary salt was reported in the form of sodium. The objective of this review was to identify daily dietary salt intake instead of dietary sodium, thus, to maintain consistency in reporting, we converted dietary and urinary sodium values to salt intake expressed/estimated in g/day using the same formula. We used these formulae to convert the sodium to salt.: 1 mmol Na = 1 mEq Na; 1 mEq Na = 23 mg Na; 1000 mg Na (1 g Na) = 2.54 g salt [15].

## 3. Results

### 3.1. Selected Studies

Using the pre-determined search terms, 1940 articles were identified through the published database and 100 articles from the grey literature database. Two survey reports were also included from the WHO STEPwise approach to the NCD Risk Factor Surveillance (STEPS)’s official website [16]. Additionally, one unpublished report was included from personal contact with the researcher, for a total of 2043 records. After 711 duplicate entries were removed, 1332 articles were reviewed using the inclusion and exclusion criteria. Following the title screening, 1064 articles were excluded. During the abstract screening, 220 of the 268 articles were eliminated. The remaining 48 records were given a complete text evaluation, with 12 of them meeting the study’s requirements and being included in the review (Figure 1).

### 3.2. Characteristics of the Included Studies

Our scoping review of salt intake includes one study from Afghanistan (n = 1), Bhutan (n = 1), Pakistan (n = 1), and Sri Lanka (n = 1), two studies from Nepal (n = 2), and three studies from Bangladesh (n = 3) and India (n = 3). Nationally representative research was available in Afghanistan, Bangladesh, Bhutan, Nepal, and India, but was absent for Pakistan and Sri Lanka. We were unable to find any studies for the Maldives. Six studies included in this review estimated salt intake by using spot urine and six studies used 24 h urine samples. (Table 4A,B shows the characteristics of the studies included in our study, and Table 5A,B shows the salt level of the included study.)

### 3.3. Countries with National Salt Intake Estimates

**Figure 2:**
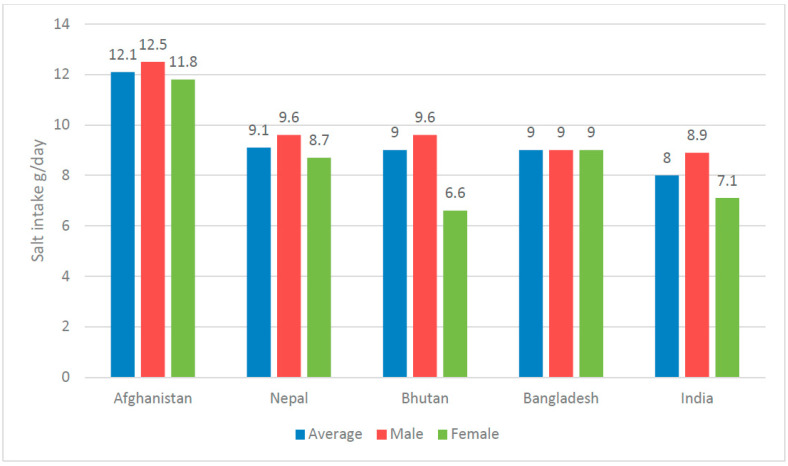
Reported amount of salt intake in nationally representative studies by country and gender.

Based on nationally representative studies, we noted that Afghanistan had the highest average salt consumption in this region (12.1 g/day) [23], followed by Nepal (9.1 g/day) [28], Bhutan (9 g/day) [25], Bangladesh (9 g/day) [24], and India (8 g/day) [26]. All those studies used the spot urine methods to estimate sodium excretion. In all countries, men consume more salt than women (Figure 2 shows the distribution of salt intake according to gender).

### 3.4. Salt Intake in Available Studies of South Asia

In our review, we found salt intake varies from country to country in the South Asian region. In the reviewed seven countries of South Asia, we included 12 studies. In the South Asian region, the salt intake levels, as determined by 24 h urine, range from 6.7 g/day (in Bangladesh) [17] to 13.3 g/day (in Nepal) [20]. On the other hand, estimating using spot urine, the salt intake ranges from 6.7 g/day (in India) [27] to 12.1 g/day (in Afghanistan) [23]. The level of the salt intake of the reviewed countries is given in Table 5A,B.

#### 3.4.1. Afghanistan

In this review, we identified a nationally representative STEPS survey report of Afghanistan [23]. The INTERSALT equation was used in the survey to estimate the population’s 24 h salt intake based on sodium levels in spot urine samples. The average salt intake was 12.1 g/day. Afghani men consumed more salt (12.5 g/day) than women (11.8 g/day). Almost one-third (32.4%) of the population added salt before eating or during eating, and almost all the people (98%) used salt during cooking. In total, 12.1% of participants regularly or always eat processed meals high in salt, and most were between 15 and 29 years old.

#### 3.4.2. Bangladesh

Based on our criteria, two published articles, and one unpublished report from Bangladesh were included in our review. Among the included studies, two used spot urine samples, and one used 24 h urine samples. Salt intake in the Bangladeshi population ranged from 6.7 g/day [17] to 9 g/day [18,24]. According to the STEPS survey, which used the spot urinary sampling method, dietary salt intake (9 g/day) was 1.8 times above the limit [24]. Almost half (48.2%) of the population added salt before or during eating, 1.8% of respondents in the survey said they frequently or always add salty sauce, and 13.5% of people consume salty processed foods on a regular basis. Using a single 24 h urine sample, Rasheed et al. [17] determined that the average daily salt intake was 6.7 g/day in Chakaria, a coastal district of Bangladesh. Another study, conducted by Choudhury et al. [18] in three divisions of Bangladesh using a 24 h urine sample, found the salt intake was 9 g/day.

#### 3.4.3. Bhutan

Under this review, we explored Bhutan’s nationally representative STEPS survey [25]. The INTERSALT equation was used to calculate 24 h urinary salt using spot urine. Bhutanese people consumed almost double (9 g/day) the sodium compared to the WHO recommendations. In total, 41.6% of the population reported that they often or always added salt to their meals and 11.1% of the population said they ate salty processed foods. In urban regions (18.8%), processed foods are consumed more frequently than in rural areas (7.5%).

#### 3.4.4. India

A nationwide representative study, a multistate study (Delhi and Haryana from north India, and Andhra Pradesh from south India), and a third study from South India were all included in this review based on the predetermined criteria. The reported dietary salt intake in the studies ranged from 6.7 g/day [27] to 9.5 g/day [19]. In 2017–2018, the Countrywide NCD Monitoring Study (NNMS) performed a national cross-sectional survey, where the average salt consumption was 8 g/day, and 15.1% of the population added salt frequently or constantly before or during eating [26]. Johnson et al. [19] estimated salt intake using a 24 h urinary sample and found that the average salt intake was 8.6 g/day in Delhi and Haryana and 9.5 g/day in Andhra Pradesh. The study conducted by Sarma et al. [27] found that the daily salt intake was 6.7 g/day using spot urine samples in the South Indian population.

#### 3.4.5. Nepal

We found two studies from Nepal that assessed salt intake; one used 24 h urinary samples conducted by Neupane et al. [20], and another one used spot urine samples which was a STEPS survey [28]. The STEPS survey found that the daily population salt consumption was 9.1 g/day, and the highest salt intake (9.4 g/day) was reported in the younger age group (25–39 years). In total, 4.5% of respondents said they used salty sauces, and 5.6% said they added salt before or while eating. In addition, 19.5% regularly or always ate processed foods rich in salt [28]. Neupane et al. conducted a study in the Kaski district of Western Nepal where the estimated daily salt intake was 13.3 g/day and was significantly associated with the male gender and younger age group [20].

#### 3.4.6. Pakistan

A study conducted by Saqib et al. [21] in Islamabad using a 24 h urinary sample found the salt intake was 8.4 g/day, which was 1.5 times higher than the WHO recommended maximum daily intake level, and females consumed more salt than males. That study also reported an association of discretionary salt usage with the male gender (*p* < 0.004) and adding salt during cooking (*p* < 0.0001). A majority (71%) of the participants were using salt in their daily food while more than half were also adding extra salt at the dining table.

#### 3.4.7. Sri Lanka

Our analysis discovered one study from Sri Lanka that estimated daily salt intake. We were unable to locate any nationally representative study. Using a 24 h urinary sample, Jayatissa et al. found that the salt intake was 8.4 g/day which was significantly higher in males (9.0 g/day) than females (7.7 g/day) [22].

## 4. Discussion

In this review, we compiled reports on population-based salt intakes in South Asian countries. Our review presents a comprehensive view of the reliably measured estimates of salt intakes in South Asian populations. To the best of our knowledge, we tried to include the study estimating sodium using the 24 h urine and spot urine, and also tried to discuss the nationally representative studies. The findings from this review suggest that the reports on the salt intake of South Asian countries varied widely between 6.7 g/day [17,27] to 13.3 g/day [20]. Using the 24 h urinary method salt intake ranges from 6.7 g/day (in Bangladesh) [17] to 13.3 g/day (in Nepal) [20], and using the spot urinary method, salt intake ranges from 6.7 g/day (in India) [27] to 12.1 g/day (in Afghanistan) [23]. Considering the nationally representative studies only, salt intake in South Asian countries ranges from 8 g/day [26] to 12.1 g/day [23]. Regardless of the salt estimation methods and study representativeness, salt intake in South Asian countries is much higher than the WHO-recommended maximum intake. The intake level is fairly comparable to the findings of the previous reviews conducted on Southeast Asian nations, which range from 6.5 g/day to 9.9 g/day. A high daily salt intake is also common in developed countries such as Europe where the salt intake ranges from 6 g/day to 15 g/day [29], and is nearly double the WHO-recommended limit in Australia (9.0 g/day) [30] and in US adults (9.2 g/day) [31].

In every country except Pakistan, men consumed more salt than women did, according to our findings. All the nationally representative studies in the review reported that men consumed more salt than women. This aligns with reviews by Kwong et al. [29] and Batcagan-Abueg et al. [32] that looked at salt consumption in Europe and Southeast Asia, respectively. It has been suggested that men’s higher salt intake is attributable to their higher overall food intake when compared to women. Another explanation might be eating out of home, which is more common in males than in women, given that food prepared outside the home is often higher in salt than home-cooked foods [33].

Having an accurate assessment of the salt consumption levels in a population is useful for planning, advocating for, and monitoring salt reduction strategies. Not having nationally representative information about the population’s salt intake can impede the formation of salt reduction initiatives, and more nationally representative studies using 24 h urine collections are recommended for countries lacking this data. However, this study demonstrates that salt intake is high, consistently above the WHO recommendations across South Asian countries. Therefore, even where nationally representative studies do not exist, salt reduction strategies can still be recommended and countries in the region should not delay efforts to develop policies and strategies to lower the salt consumption of their countries.

Given the negative effects of excessive salt consumption, 194 member states of WHO committed to reducing the population’s salt intake by 30%. Reducing salt in commonly consumed foods is an efficient method of salt reduction. While many developed nations have distinct policies in place to reduce salt consumption, particularly in processed packaged foods, few low-and middle-income countries have developed policies, despite the increasing consumption in most countries. Setting sodium targets to mandate the reformulation of processed packaged food is a simple and effective way to reduce the population’s daily sodium consumption. In total, 34% of WHO member states have implemented policies to reformulate processed food to reduce the sodium content, and 28% of states have public food procurement and service policies. Nutritional labeling, particularly front-of-pack labeling (FOPL) of pre-packaged foods is an effective tool that enables consumers to make healthier choices and may help them to avoid sodium-rich foods. In addition, 21% of member states have implemented FOPL. Sri Lanka has a mandatory FOPL system to reduce the growing burden of NCD. Bangladesh and India have a policy of the mandatory declaration of sodium in pre-packaged foods; the Maldives and Nepal only have a national policy commitment to reduce sodium. Further efforts, including policy development, are urgently required by South Asian countries’ governments, along with sustained leadership and a strong commitment to sodium reduction. Multi-sectoral involvement is needed to achieve population-wide sodium reduction. Civil society and media personnel can help in creating population awareness, but food industries must ensure compliance on their part, with government agencies and academia monitoring implementation.

This study has a few limitations We were unable to identify relevant publications, studies, or statistics on salt intake in the Maldives. Further, data were not pooled for meta-analysis. Our review is confined to articles published in English, so we may have missed additional relevant studies in local languages. To our knowledge, we included the most recent studies between January 2011 and October 2021. One of this study’s strongest features is that it contained a thorough search of all the literature on the population’s salt intake in the region, including both individual and nationally representative research, and we only included studies that estimated salt intake using 24 h or spot urine samples.

## 5. Conclusions

Information on salt intake in South Asia is limited. Afghanistan, Bangladesh, Bhutan, India, and Nepal have nationally representative data on population-level salt intake, while Pakistan and Sri Lanka’s salt consumption data are not nationally representative. None of the nationally representative studies estimated salt intake using the standard 24 h urinary excretion method. The salt estimation techniques of the studies included in this review varied. Thus, it is difficult to confirm whether differences in the level of salt intake are reflected by different dietary habits or because of the differences in methodological approaches. Studies of this review suggest that salt intake levels in all countries remain higher than the WHO-recommended maximum level of daily intake. To lessen the burden of NCDs linked with a high salt intake, the government must urgently prioritize implementing them throughout the nations of the South Asian region and ensure a food environment in which people may eat healthy, nutritious meals with a minimal salt content, and countries should develop comprehensive national sodium reduction strategies following WHO recommendations found in the WHO SHAKE Technical Package. This strategy will guide government agencies to develop impactful policies and programs to reduce and monitor salt intake. Strategies should address out-of-home environments through healthy public food procurement and service policies, salt consumed at the home through raising awareness via mass media campaigns and through the promotion of low-sodium salt, and the packaged food environment, through policies including front-of-package labeling, marketing restrictions, and mandatory sodium targets. Regional sodium benchmarks for packaged foods are currently being finalized by the WHO SEARO office, which will be a useful step in supporting countries to set national targets to reduce salt levels in processed and packaged foods to lower the population’s salt intake.

## Figures and Tables

**Table 1:** List of databases searched.

For Published Article	For the Grey Literature
PubMed	Open grey
MEDLINE	Google Scholar
EMBASE	WHO official database
EBSCO	Key informant consultation
	Country-specific Government website

**Table 2:** Search terms used in the scoping review.

Topic	Search Terms
Salt	Salt OR Sodium OR Sodium Chloride
Consumption	Intake OR Ingest OR Eat OR Consume OR Diet
Excretion	Urine OR Excrete OR 24 h urine OR Spot urine
South Asia	Asia OR South Asia OR Afghanistan OR Bangladesh OR India OR Nepal OR Pakistan OR Sri Lanka OR Bhutan OR Maldives
Search string: (Salt OR Sodium OR Sodium Chloride) AND (Intake OR Ingest OR Eat OR Consume OR Diet) AND (Urine OR Excrete OR 24 h urine OR Spot urine) AND (Asia OR South Asia OR Afghanistan OR Bangladesh OR India OR Nepal OR Pakistan OR Sri Lanka OR Bhutan).	

**Table 3:** Inclusion and exclusion criteria of study selection.

Inclusion Criteria	Exclusion Criteria
1. Studies conducted in SAARC countries (Afghanistan, Bangladesh, India, Nepal, Pakistan, Sri Lanka, and Bhutan)	1. If the salt intake was not reported in standard statistics (mean/median intake of salt)
2. Studies estimated salt/sodium intake by measuring urinary sodium excretion	2. Studies conducted on only special sub-populations
3. Studies conducted among minimum of 100 participants	3. Studies conducted among pregnant women or children
4. Written in the English language	
5. Published between January 2011 to October 2021	

**Table 4:** (**A**) Characteristics of studies that examined salt intake using 24 h urine. (**B**) Characteristics of studies that examined salt intake using spot urine.

(**A**)					
**Country**	**Authors**	**Year**	**Type of Study**	**Sampling Technique, Study Population, Sample Size, and Sample Characteristics (Age and Sex)**	
Bangladesh	Rasheed et al. [17]	2014	Cross-sectional study	388 individuals from a community were randomly chosen for a cross-sectional household survey in Chakaria, a rural area of Bangladesh. The participants’ mean age was 44.6 years, with 50% of them being female.	
Bangladesh	Choudhury et al. [18]	-	Cross-sectional study	839 community members were randomly chosen from the urban and rural areas of Khulna, Dhaka, and Rangpur, aged 30–59 years, and 49.8% were female.	
India	Johnson et al. [19]	2017	Cross-sectional study	637 adults, the average age was 40.2 years and 48.2% were female selected from urban, rural, and slum areas in north India (Delhi and Faridabad, Haryana) and in south India (Hyderabad and West Godavari, Andhra Pradesh).	
Nepal	Neupane et al. [20]	2019	Cross-sectional study	451 randomly selected participants, with a mean age of 49.6 years, and 65.4% were female.	
Pakistan	Saqib et al. [21]	2020	Cross-sectional study	120 participants were chosen using a non-probability convenient sampling method; the average age was 26.5 years, and 23% were female.	
Sri Lanka	Jayatissa et al. [22]	2020	Cross-sectional study	328 randomly selected community residents aged 30–59 years, with 53.7% being female.	
(**B**)					
**Country**	**Authors**	**Year**	**Type of Study**	**Sampling Technique, Study Population, Sample Size, and Sample Characteristics (Age and Sex)**	**Equation Used for Estimating Sodium Intake Using Spot Urine**
Afghanistan	WHO [23]	2018	Population-based national cross-sectional STEPS study	3956 community dwellers, aged 18–69 years and 48.8% female were selected by multistage stratified random sampling technique.	INTERSALT
Bangladesh	WHO [24]	2018	Population-based national cross-sectional STEPS study	9900 individuals aged 18–69 years and 50.7% female were selected by a multistage geographically stratified probability-based sampling process.	Tanaka
Bhutan	WHO [25]	2014	Population-based national cross-sectional STEPS study	2816 individuals aged 18–69 years and 61.9% were female selected by multistage cluster sampling.	INTERSALT
India	Mathur et al. [26]	2021	Nationally representative cross-sectional study	2266 participants aged 18 to 69 years and 48.2% female, were chosen using a stratified multistage selection technique.	INTERSALT
India	Sarma et al. [27]	2019	Community-based cross-sectional study	12,012 participants were selected by the multistage cluster sampling method. Participants were aged 18–69 years, and 62.8% were female.	Modified Kawaski
Nepal	WHO [28]	2019	Population-based cross-sectional STEPS study	5593 adults selected through multistage cluster sampling technique, aged 55–69 years, and 63.3% were female	INTERSALT

**Table 5:** (**A**) Studies reporting salt intake by estimating 24 h urine in South Asian countries. (**B**) Studies reporting salt intake by estimating spot urine in South Asian countries.

Country	Authors and Year	Level of Salt Intake (g/day)		
		Average	Male	Female
(**A**)				
Bangladesh	Rasheed et al. (2014) [17]	6.7	-	-
	Choudhury [18]	9.0 (9 ± 4.3)	-	-
India	Johnson et al. (2017) [19]	Andhra Pradesh: 9.5 (9.1–9.9) Delhi and Haryana: 8.6 (7.9–9.5)	-	-
Nepal	Neupane et al. (2019) [20]	13.3 (12.8–13.7)	14.4 (13.6–15.2)	12.7 (12.2–13.2)
Pakistan	Saqib et al. (2020) [21]	8.7 (4.2–13.1)	9.2 (4.7–13.8)	8.7 (4.2–13.1)
Sri Lanka	Jayatissa et al. (2020) [22]	8.4 (7.9–8.8)	9.0 (8.3–9.8)	7.7 (7.2–8.2)
(**B**)				
Afghanistan	WHO (2018) [23]	12.1 (11.1–13.1)	12.5 (10.9–14)	11.8 (10.5–13.1)
Bangladesh	WHO (2018) [24]	9 (8.9–9.1)	12.5 (10.9–14.0)	11.8 (10.5–13.1)
Bhutan	WHO (2014) [25]	9 (8.8–9.1)	9.6(9.4–9.8)	8 (7.9–8.2)
India	Mathur et al. (2021) [26]	8.0 (7.8–8.2)	8.9 (8.7–9.2)	7.1 (6.9–7.2)
	Sarma et al. (2019) [27]	6.7 (6.6–6.8)	5.3 (5.2–5.4)	7.5 (7.4–7.6)
Nepal	WHO (2019) [28]	9.1 (9.0–9.2)	9.6 (9.4–9.8)	8.7 (8.6–8.8)

## Data Availability

Not applicable.
